# Crystal structure of bis­(2-{[(3-bromo­prop­yl)imino]­meth­yl}phenolato-κ^2^ *N*,*O*)copper(II)

**DOI:** 10.1107/S2056989015001309

**Published:** 2015-01-24

**Authors:** Ali Ourari, Chahinaz Zoubeidi, Sofiane Bouacida, Wassila Derafa, Hocine Merazig

**Affiliations:** a Laboratoire d’Electrochimie, d’Ingénierie Moléculaire et de Catalyse Redox (LEIMCR), Faculté des Sciences de l’Ingénieur, Université Farhat Abbas, Sétif 19000, Algeria; b Département Sciences de la Matière, Faculté des sciences Exactes et Sciences de la Nature et de la Vie, Université Oum El Bouaghi, Algeria; c Unité de Recherche de Chimie de l’Environnement et Moléculaire Structurale, CHEMS, Université Constantine 1, 25000 , Algeria

**Keywords:** crystal structure, copper(II) complex, C—H⋯π inter­actions

## Abstract

In the title compound, [Cu(C_10_H_11_BrNO)_2_], the asymmetric unit consists of one-half of the mol­ecule, the other half being generated by an inversion centre. Hence the Cu^II^ cation is symmetrically coordinated by two bidentate Schiff base anions in a slightly distorted square-planar environment with Cu—O and Cu—N bond lengths of 1.8786 (19) and 2.009 (2) Å, respectively. In the crystal, individual mol­ecules are packed in alternating zigzag layers parallel to (001). Weak C—H⋯π inter­actions exist between the mol­ecules.

## Related literature   

For synthesis and applications of similar complexes derived from salicyl­aldehyde, see: Ghelenji *et al.* (2011 ▸); Kia *et al.* (2010 ▸); Zhang *et al.* (2013 ▸). For the importance of copper in biological systems, see: Siegel (1973 ▸); Mohan *et al.* (1998 ▸). For isotypic structures, see: Floyd *et al.* (2005 ▸); Ourari *et al.* (2015 ▸).
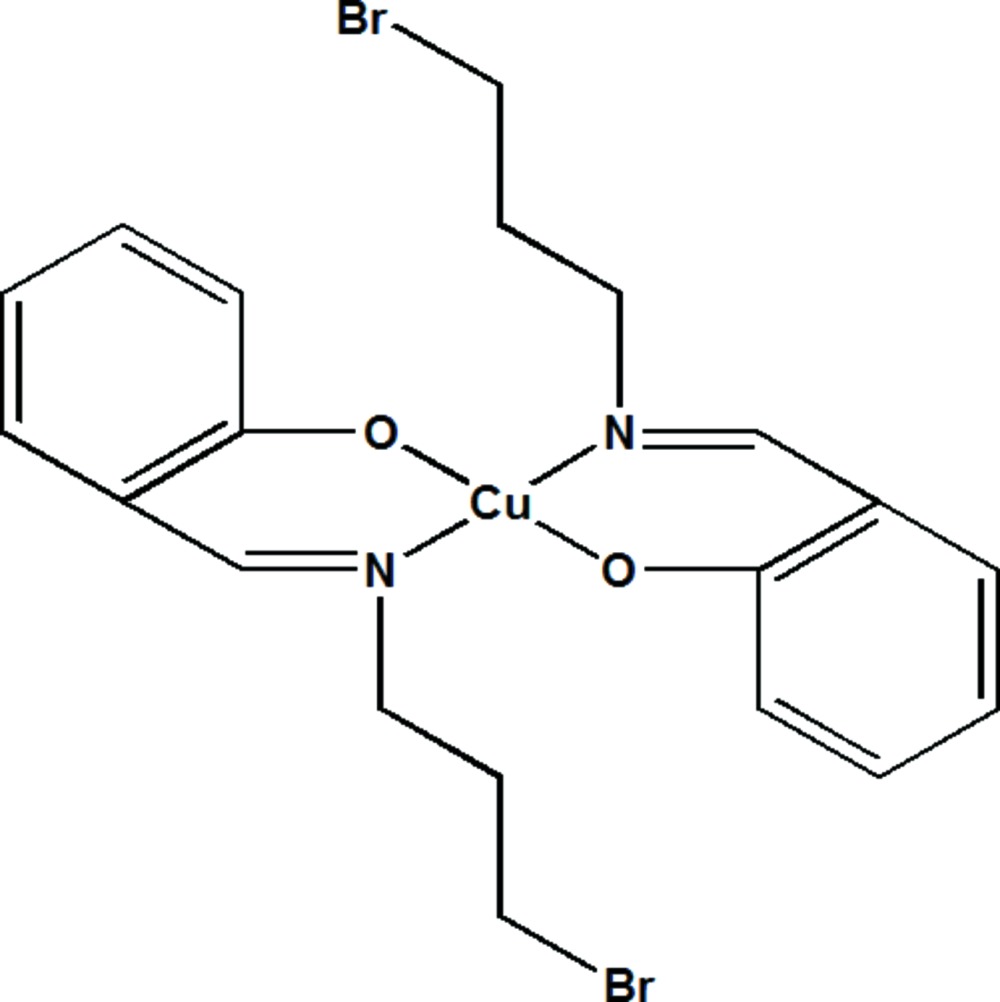


## Experimental   

### Crystal data  

[Cu(C_10_H_11_BrNO)_2_]
*M* *_r_* = 545.75
Monoclinic, 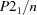

*a* = 10.6478 (4) Å
*b* = 7.1990 (3) Å
*c* = 13.9283 (5) Å
β = 104.900 (2)°
*V* = 1031.75 (7) Å^3^
*Z* = 2
Mo *K*α radiation
μ = 4.95 mm^−1^
*T* = 295 K
0.19 × 0.18 × 0.15 mm

### Data collection  

Bruker APEXII diffractometer
Absorption correction: multi-scan (*SADABS*; Bruker, 2011 ▸) *T*_min_ = 0.677, *T*_max_ = 0.796
8212 measured reflections
2594 independent reflections
2088 reflections with *I* > 2σ(*I*)
*R*_int_ = 0.020

### Refinement  

*R*[*F* ^2^ > 2σ(*F* ^2^)] = 0.032
*wR*(*F* ^2^) = 0.089
*S* = 1.04
2594 reflections
124 parameters
H-atom parameters constrained
Δρ_max_ = 0.62 e Å^−3^
Δρ_min_ = −0.58 e Å^−3^

### 

Data collection: *APEX2* (Bruker, 2011 ▸); cell refinement: *SAINT* (Bruker, 2011 ▸); data reduction: *SAINT*; program(s) used to solve structure: *SIR2002* (Burla *et al.*, 2005 ▸); program(s) used to refine structure: *SHELXL97* (Sheldrick, 2015 ▸); molecular graphics: *ORTEP-3 for Windows* (Farrugia, 2012 ▸) and *DIAMOND* (Brandenburg, 2001 ▸); software used to prepare material for publication: *WinGX* (Farrugia, 2012 ▸).

## Supplementary Material

Crystal structure: contains datablock(s) I. DOI: 10.1107/S2056989015001309/wm5116sup1.cif

Structure factors: contains datablock(s) I. DOI: 10.1107/S2056989015001309/wm5116Isup2.hkl

**Fig. 1.**
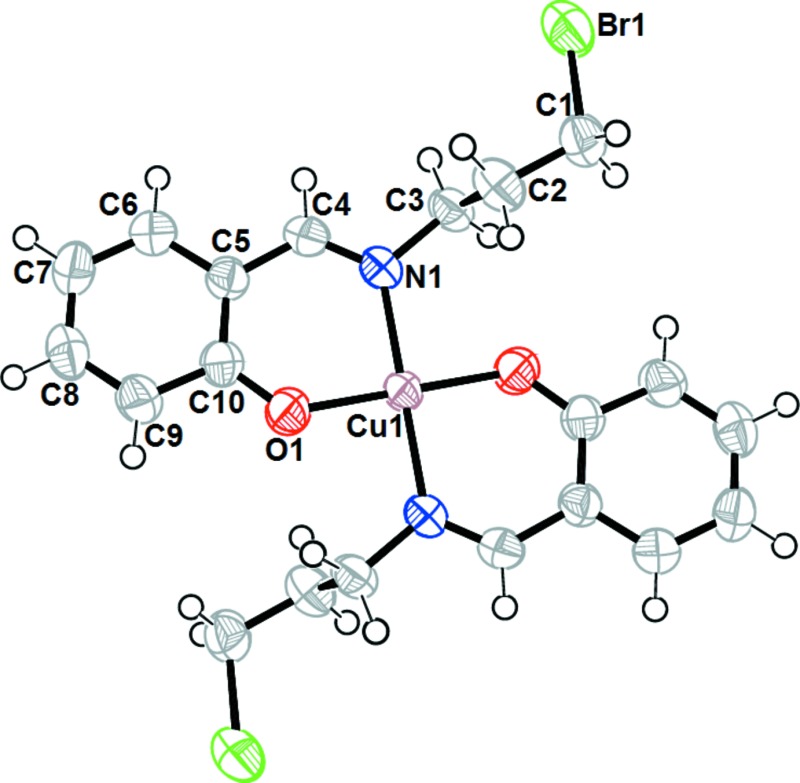
The molecular structure of the title compound with the atomic labelling scheme. Displacement are drawn at the 50% probability level. Non-labelled atoms are generated by symmetry code -x+2, -y, -z+2.

. DOI: 10.1107/S2056989015001309/wm5116fig1.tif
The mol­ecular structure of the title compound with the atomic labelling scheme. Displacement are drawn at the 50% probability level. Non-labelled atoms are generated by symmetry code −x+2, −y, −z+2.

**Fig. 2.**
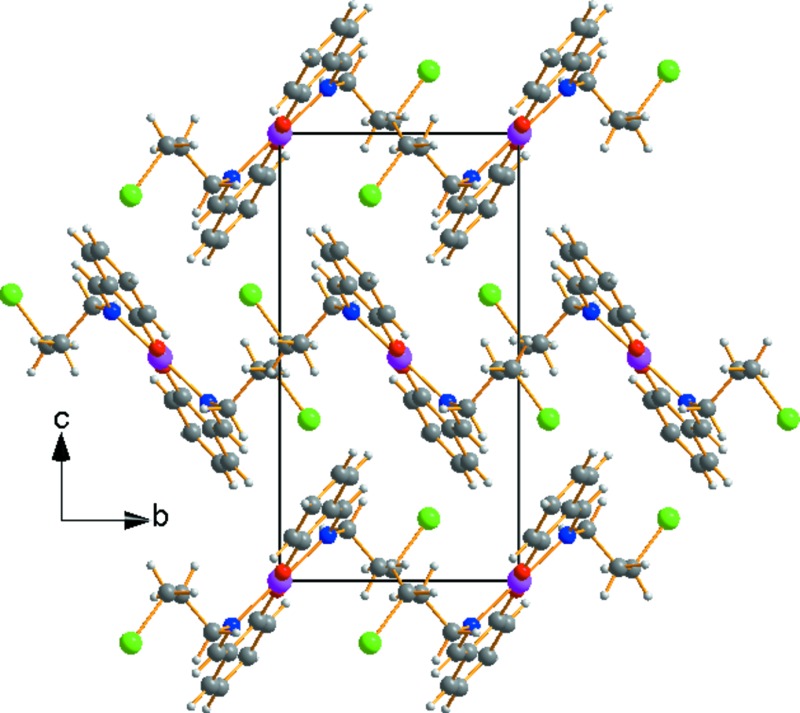
Formation of alternating zigzag layers parallel to (001).

. DOI: 10.1107/S2056989015001309/wm5116fig2.tif
Formation of alternating zigzag layers parallel to (001).

**Fig. 3.**
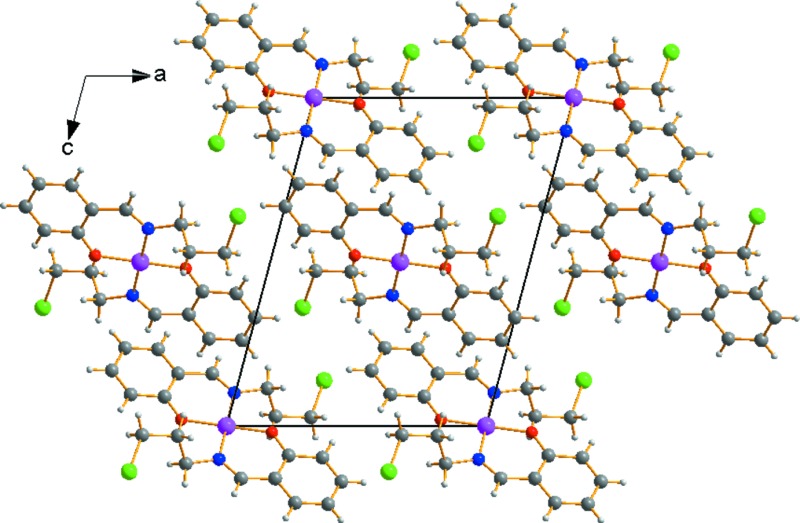
A view of the layers along [010].

. DOI: 10.1107/S2056989015001309/wm5116fig3.tif
A view of the layers along [010].

CCDC reference: 1044698

Additional supporting information:  crystallographic information; 3D view; checkCIF report

## Figures and Tables

**Table 1:** Hydrogen-bond geometry (, ) *Cg*1 is the centroid of the C5C10 ring.

*D*H*A*	*D*H	H*A*	*D* *A*	*D*H*A*
C1H1*A* *Cg*1^i^	0.97	2.74	3.645(4)	155
C4H4*Cg*1^ii^	0.93	2.90	3.805(3)	164

Symmetry codes: (i) 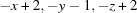; (ii) 
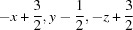
.

## supplementary crystallographic information

### S1. Experimental

Ligand (H*L*) synthesis: 331.5 mg (1.5 mmol) of 2-bromopropyl ammonium hydrobromide were dissolved in absolute ethanol (15 ml). First, 756 mg (1.5 mmol; excess of 5%) and then 183 mg salicylaldehyde, each dissolved in 10 ml of absolute ethanol, were added and the resulting solution was refluxed under nitrogen atmosphere for 2 h at 333 K. The solvent was removed under reduced pressure and 15 ml of dichloromethane were added to the residue obtained. The mixture was stirred for 15 min, filtered and the solvent evaporated, resulting in a yellow viscous oil (yield: 82%).

Synthesis of the copper complex (I): 215 mg ligand H*L* (1 mmol) were placed in 10 ml of absolute ethanol. 99.8 mg of copper acetate monohydrate (0.5 mmol), dissolved in 5 ml of absolute ethanol, were added to this solution. The content of the flask was refluxed under stirring and nitrogen atmosphere for 2 h at 333 K. The precipitate obtained was filtered, washed with ethanol and then dried in an oven at moderate temperature (yield 70%; m. p. 393 K). Suitable single crystals were obtained from acetone solution by slow evaporation yielding green single crystals.

### S2. Refinement

H atoms were localized in Fourier maps but introduced in calculated positions and treated as riding on their parent atom, with C—H = 0.97 Å (methylene) or 0.93 Å (aromatic) and with *U*_iso_(H) = 1.2*U*_eq_. Reflection 101 was obstructed from the beam stop and was omitted from the refinement.

### Crystal data

**Table:**

[Cu(C_10_H_11_BrNO)_2_]	*F*(000) = 542.0
*M**_r_* = 545.75	*D*_x_ = 1.757 Mg m^−^^3^
Monoclinic, *P*2_1_/*n*	Mo *K*α radiation, λ = 0.71073 Å
*a* = 10.6478 (4) Å	Cell parameters from 3645 reflections
*b* = 7.1990 (3) Å	θ = 3.2–27.7°
*c* = 13.9283 (5) Å	µ = 4.95 mm^−^^1^
β = 104.900 (2)°	*T* = 295 K
*V* = 1031.75 (7) Å^3^	Prism, green
*Z* = 2	0.19 × 0.18 × 0.15 mm

### Data collection

**Table:**

Bruker APEXII diffractometer	2088 reflections with *I* > 2σ(*I*)
Graphite monochromator	*R*_int_ = 0.020
CCD rotation images, thin slices scans	θ_max_ = 28.5°, θ_min_ = 2.8°
Absorption correction: multi-scan (*SADABS*; Bruker, 2011)	*h* = −12→14
*T*_min_ = 0.677, *T*_max_ = 0.796	*k* = −9→6
8212 measured reflections	*l* = −18→18
2594 independent reflections	

### Refinement

**Table:**

Refinement on *F*^2^	Primary atom site location: structure-invariant direct methods
Least-squares matrix: full	Secondary atom site location: difference Fourier map
*R*[*F*^2^ > 2σ(*F*^2^)] = 0.032	Hydrogen site location: inferred from neighbouring sites
*wR*(*F*^2^) = 0.089	H-atom parameters constrained
*S* = 1.04	*w* = 1/[σ^2^(*F*_o_^2^) + (0.0419*P*)^2^ + 0.8205*P*] where *P* = (*F*_o_^2^ + 2*F*_c_^2^)/3
2594 reflections	(Δ/σ)_max_ = 0.001
124 parameters	Δρ_max_ = 0.62 e Å^−^^3^
0 restraints	Δρ_min_ = −0.58 e Å^−^^3^

### Special details

**Table:**

Geometry. All e.s.d.'s (except the e.s.d. in the dihedral angle between two l.s. planes) are estimated using the full covariance matrix. The cell e.s.d.'s are taken into account individually in the estimation of e.s.d.'s in distances, angles and torsion angles; correlations between e.s.d.'s in cell parameters are only used when they are defined by crystal symmetry. An approximate (isotropic) treatment of cell e.s.d.'s is used for estimating e.s.d.'s involving l.s. planes.
Refinement. Refinement of *F*^2^ against ALL reflections. The weighted *R*-factor *wR* and goodness of fit *S* are based on *F*^2^, conventional *R*-factors *R* are based on *F*, with *F* set to zero for negative *F*^2^. The threshold expression of *F*^2^ > σ(*F*^2^) is used only for calculating *R*-factors(gt) *etc*. and is not relevant to the choice of reflections for refinement. *R*-factors based on *F*^2^ are statistically about twice as large as those based on *F*, and *R*- factors based on ALL data will be even larger.

### Fractional atomic coordinates and isotropic or equivalent isotropic displacement parameters (Å^2^)

**Table:**

	*x*	*y*	*z*	*U*_iso_*/*U*_eq_	
Cu1	1	0	1	0.03472 (12)	
Br1	1.32859 (3)	−0.62235 (6)	0.86101 (3)	0.06803 (14)	
O1	0.81891 (18)	−0.0114 (3)	0.98050 (16)	0.0559 (6)	
N1	0.99577 (19)	−0.2004 (3)	0.89887 (15)	0.0350 (4)	
C5	0.7594 (2)	−0.2139 (3)	0.84019 (19)	0.0368 (5)	
C8	0.5014 (3)	−0.1335 (4)	0.8310 (2)	0.0476 (7)	
H8	0.4152	−0.1059	0.8282	0.057*	
C4	0.8909 (2)	−0.2635 (4)	0.84046 (19)	0.0382 (5)	
H4	0.9006	−0.3503	0.7934	0.046*	
C9	0.5984 (3)	−0.0546 (4)	0.9037 (2)	0.0468 (6)	
H9	0.5767	0.0251	0.9494	0.056*	
C6	0.6576 (3)	−0.2913 (4)	0.7663 (2)	0.0492 (7)	
H6	0.677	−0.3702	0.7192	0.059*	
C3	1.1185 (2)	−0.2808 (4)	0.88563 (19)	0.0394 (5)	
H3A	1.1795	−0.1817	0.8837	0.047*	
H3B	1.1011	−0.3469	0.8229	0.047*	
C10	0.7306 (2)	−0.0917 (4)	0.91054 (19)	0.0392 (5)	
C2	1.1783 (3)	−0.4136 (4)	0.9701 (2)	0.0471 (6)	
H2A	1.1776	−0.3551	1.0326	0.056*	
H2B	1.1249	−0.5245	0.9633	0.056*	
C7	0.5294 (3)	−0.2529 (5)	0.7621 (2)	0.0522 (7)	
H7	0.4628	−0.3068	0.7134	0.063*	
C1	1.3158 (3)	−0.4696 (4)	0.9730 (2)	0.0493 (7)	
H1A	1.3521	−0.5375	1.034	0.059*	
H1B	1.3676	−0.3584	0.9742	0.059*	

### Atomic displacement parameters (Å^2^)

**Table:**

	*U*^11^	*U*^22^	*U*^33^	*U*^12^	*U*^13^	*U*^23^
Cu1	0.0302 (2)	0.0403 (2)	0.0355 (2)	−0.00058 (17)	0.01176 (16)	−0.00451 (17)
Br1	0.0490 (2)	0.0798 (3)	0.0742 (2)	0.01763 (16)	0.01380 (16)	−0.01284 (18)
O1	0.0347 (9)	0.0754 (15)	0.0616 (12)	−0.0084 (9)	0.0195 (9)	−0.0318 (11)
N1	0.0324 (10)	0.0359 (11)	0.0380 (10)	0.0046 (8)	0.0115 (8)	0.0000 (8)
C5	0.0342 (12)	0.0326 (12)	0.0428 (13)	0.0009 (10)	0.0083 (10)	0.0000 (10)
C8	0.0324 (12)	0.0529 (17)	0.0582 (16)	0.0005 (12)	0.0129 (12)	0.0067 (13)
C4	0.0403 (12)	0.0333 (12)	0.0416 (13)	0.0047 (10)	0.0118 (10)	−0.0034 (10)
C9	0.0382 (13)	0.0504 (15)	0.0570 (16)	−0.0021 (12)	0.0213 (12)	−0.0063 (13)
C6	0.0428 (14)	0.0445 (15)	0.0576 (17)	0.0020 (12)	0.0082 (12)	−0.0117 (13)
C3	0.0372 (12)	0.0427 (14)	0.0405 (13)	0.0073 (11)	0.0141 (10)	−0.0014 (11)
C10	0.0344 (12)	0.0411 (14)	0.0443 (13)	−0.0041 (10)	0.0140 (10)	−0.0024 (11)
C2	0.0452 (15)	0.0470 (16)	0.0515 (15)	0.0099 (12)	0.0172 (12)	0.0093 (13)
C7	0.0359 (13)	0.0553 (17)	0.0590 (17)	−0.0034 (13)	0.0008 (12)	−0.0053 (14)
C1	0.0411 (14)	0.0502 (17)	0.0534 (16)	0.0057 (12)	0.0062 (12)	0.0030 (13)

### Geometric parameters (Å, º)

**Table:**

Cu1—O1^i^	1.8786 (19)	C4—H4	0.93
Cu1—O1	1.8786 (19)	C9—C10	1.412 (4)
Cu1—N1	2.009 (2)	C9—H9	0.93
Cu1—N1^i^	2.009 (2)	C6—C7	1.380 (4)
Br1—C1	1.942 (3)	C6—H6	0.93
O1—C10	1.302 (3)	C3—C2	1.522 (4)
N1—C4	1.284 (3)	C3—H3A	0.97
N1—C3	1.484 (3)	C3—H3B	0.97
C5—C6	1.404 (4)	C2—C1	1.509 (4)
C5—C10	1.408 (4)	C2—H2A	0.97
C5—C4	1.444 (3)	C2—H2B	0.97
C8—C9	1.369 (4)	C7—H7	0.93
C8—C7	1.377 (4)	C1—H1A	0.97
C8—H8	0.93	C1—H1B	0.97
			
O1^i^—Cu1—O1	180.0000 (10)	N1—C3—C2	110.9 (2)
O1^i^—Cu1—N1	88.28 (8)	N1—C3—H3A	109.5
O1—Cu1—N1	91.72 (8)	C2—C3—H3A	109.5
O1^i^—Cu1—N1^i^	91.72 (8)	N1—C3—H3B	109.5
O1—Cu1—N1^i^	88.28 (8)	C2—C3—H3B	109.5
N1—Cu1—N1^i^	180.0000 (10)	H3A—C3—H3B	108.1
C10—O1—Cu1	130.10 (17)	O1—C10—C5	123.6 (2)
C4—N1—C3	115.7 (2)	O1—C10—C9	118.8 (2)
C4—N1—Cu1	123.89 (16)	C5—C10—C9	117.6 (2)
C3—N1—Cu1	120.39 (16)	C1—C2—C3	113.5 (2)
C6—C5—C10	119.5 (2)	C1—C2—H2A	108.9
C6—C5—C4	118.0 (2)	C3—C2—H2A	108.9
C10—C5—C4	122.5 (2)	C1—C2—H2B	108.9
C9—C8—C7	121.1 (3)	C3—C2—H2B	108.9
C9—C8—H8	119.4	H2A—C2—H2B	107.7
C7—C8—H8	119.4	C8—C7—C6	118.9 (3)
N1—C4—C5	126.8 (2)	C8—C7—H7	120.5
N1—C4—H4	116.6	C6—C7—H7	120.5
C5—C4—H4	116.6	C2—C1—Br1	113.4 (2)
C8—C9—C10	121.4 (3)	C2—C1—H1A	108.9
C8—C9—H9	119.3	Br1—C1—H1A	108.9
C10—C9—H9	119.3	C2—C1—H1B	108.9
C7—C6—C5	121.4 (3)	Br1—C1—H1B	108.9
C7—C6—H6	119.3	H1A—C1—H1B	107.7
C5—C6—H6	119.3		
			
N1—Cu1—O1—C10	−13.1 (3)	Cu1—N1—C3—C2	75.6 (3)
N1^i^—Cu1—O1—C10	166.9 (3)	Cu1—O1—C10—C5	9.8 (4)
O1^i^—Cu1—N1—C4	−170.0 (2)	Cu1—O1—C10—C9	−169.8 (2)
O1—Cu1—N1—C4	10.0 (2)	C6—C5—C10—O1	−178.9 (3)
O1^i^—Cu1—N1—C3	7.98 (19)	C4—C5—C10—O1	1.0 (4)
O1—Cu1—N1—C3	−172.02 (19)	C6—C5—C10—C9	0.8 (4)
C3—N1—C4—C5	177.6 (2)	C4—C5—C10—C9	−179.4 (3)
Cu1—N1—C4—C5	−4.3 (4)	C8—C9—C10—O1	179.3 (3)
C6—C5—C4—N1	176.5 (3)	C8—C9—C10—C5	−0.3 (4)
C10—C5—C4—N1	−3.3 (4)	N1—C3—C2—C1	−168.0 (2)
C7—C8—C9—C10	0.3 (5)	C9—C8—C7—C6	−0.7 (5)
C10—C5—C6—C7	−1.2 (4)	C5—C6—C7—C8	1.1 (5)
C4—C5—C6—C7	179.0 (3)	C3—C2—C1—Br1	−67.5 (3)
C4—N1—C3—C2	−106.3 (3)		

Symmetry code: (i) −*x*+2, −*y*, −*z*+2.

### Hydrogen-bond geometry (Å, º)

Cg1 is the centroid of the C5–C10 ring.

**Table:**

*D*—H···*A*	*D*—H	H···*A*	*D*···*A*	*D*—H···*A*
C1—H1*A*···*Cg*1^ii^	0.97	2.74	3.645 (4)	155
C4—H4···*Cg*1^iii^	0.93	2.90	3.805 (3)	164

Symmetry codes: (ii) −*x*+2, −*y*−1, −*z*+2; (iii) −*x*+3/2, *y*−1/2, −*z*+3/2.
