# Supplementary figures and images for: Crystal structure of bis­(2-{[(3-bromo­prop­yl)imino]­meth­yl}phenolato-κ2 N,O)copper(II)
Source: Acta Crystallogr E Crystallogr Commun. 2015 Jan 24;71(Pt 2):m33–4. doi: 10.1107/S2056989015001309 (PMC4384597; doi:10.1107/S2056989015001309)

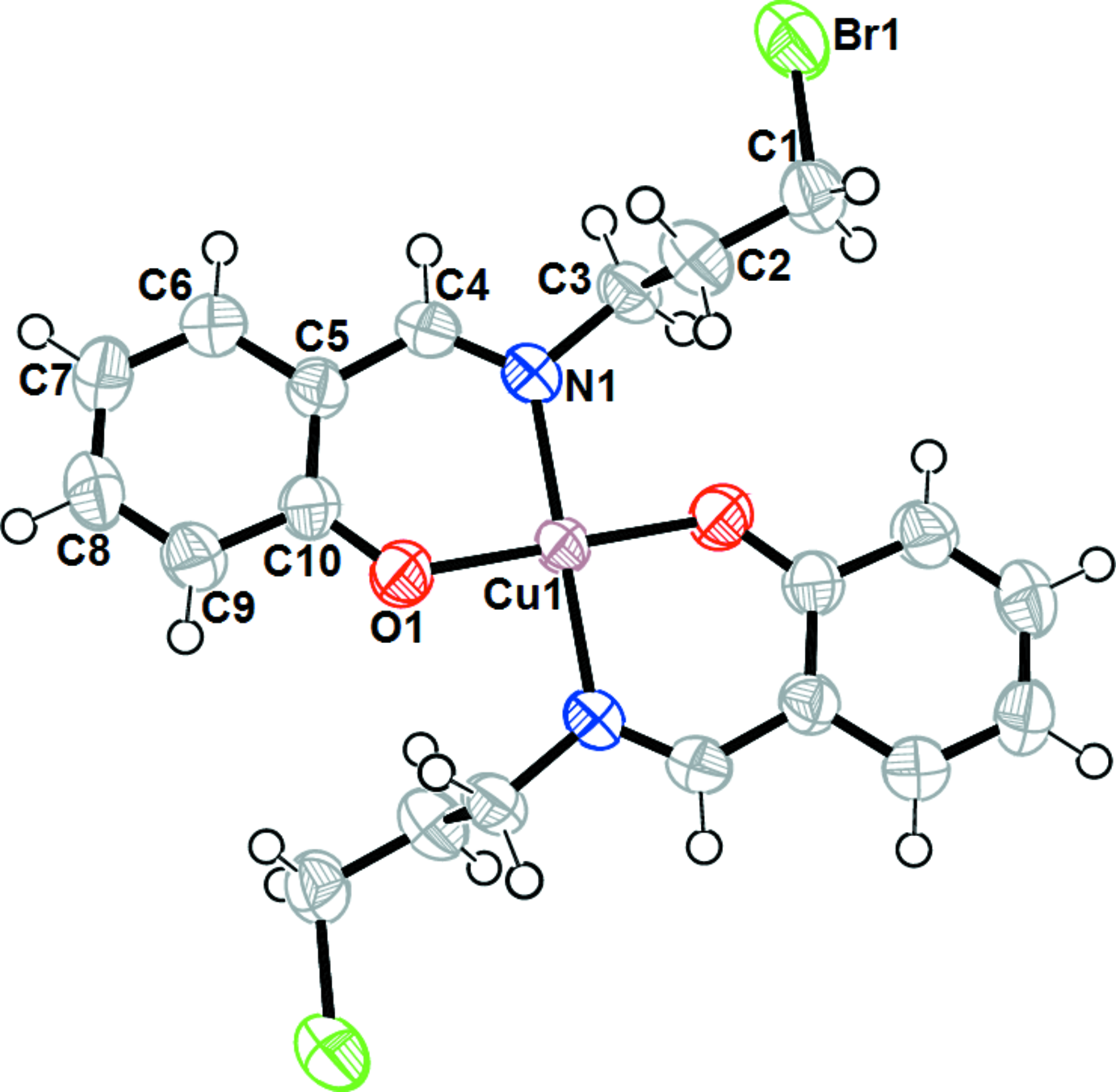

Supplement: Supplementary file 3 [file e-71-00m33-fig1.tif]

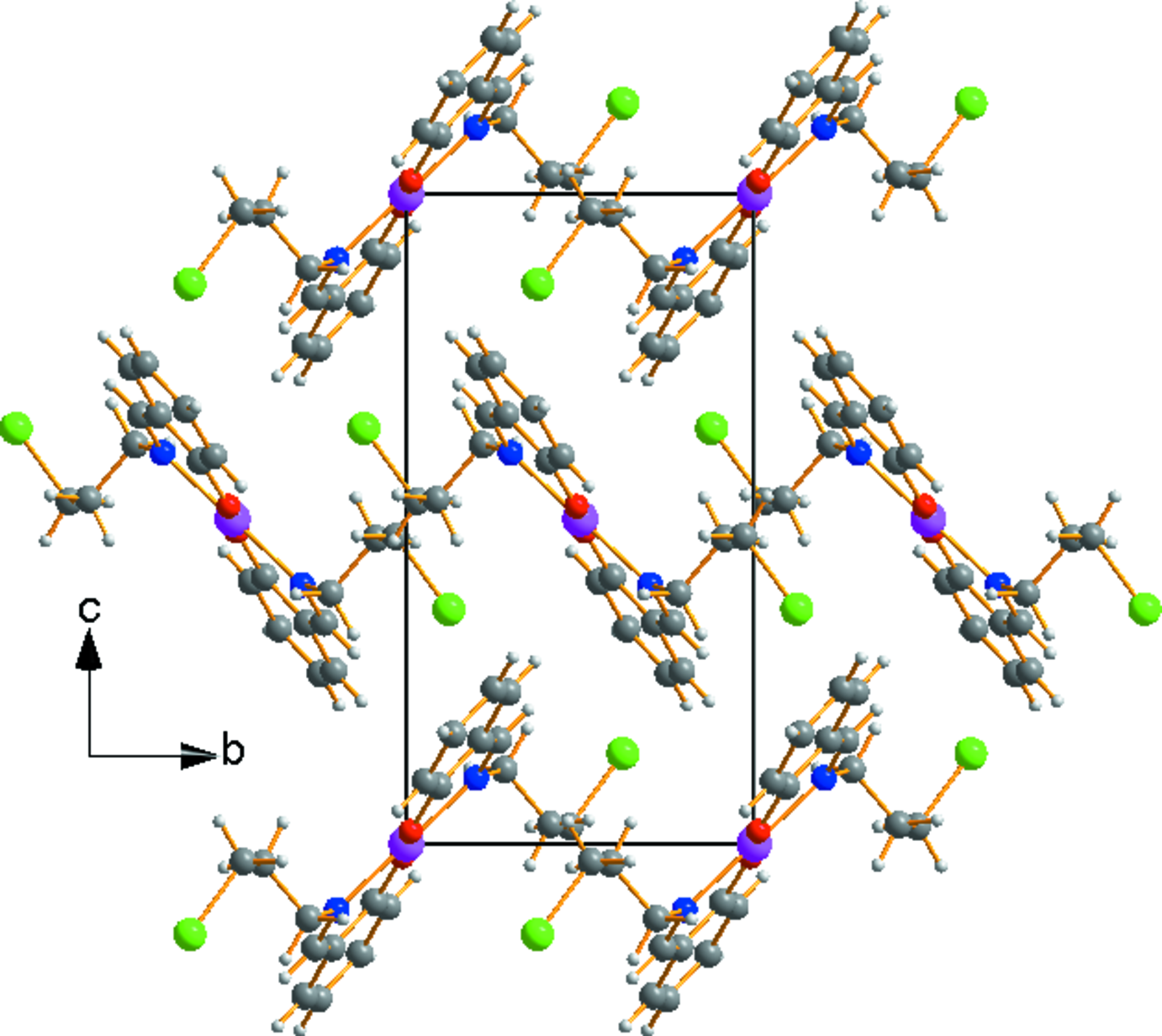

Supplement: Supplementary file 4 [file e-71-00m33-fig2.tif]

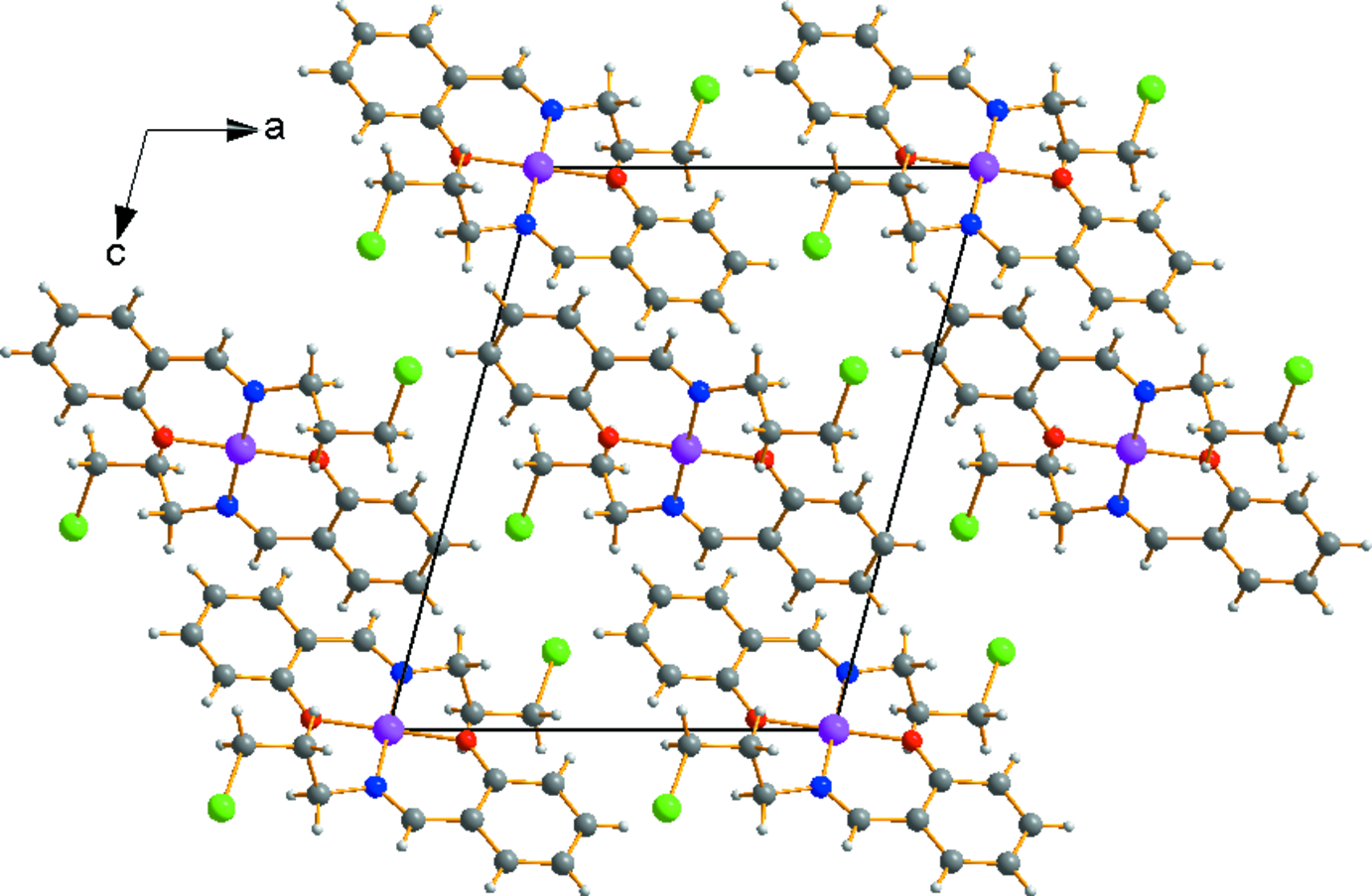

Supplement: Supplementary file 5 [file e-71-00m33-fig3.tif]
